# Real-world evidence on the safety and effectiveness of integrative Korean medicine for older patients post-traffic accident: A retrospective observational study

**DOI:** 10.1097/MD.0000000000046144

**Published:** 2025-11-28

**Authors:** Ju-Yeun Shin, Ye-Seul Lee, Seung-Hee Lee, Jeong-Hee Noh, Yoon Jae Lee, In-Hyuk Ha, Su Won Lee

**Affiliations:** a Department of Acupuncture and Moxibustion, Bundang Jaseng Hospital of Korean Medicine, Gyeonggi-do, Republic of Korea; b Jaseng Spine and Joint Research Institute, Jaseng Medical Foundation, Seoul, Republic of Korea; c Department of Korean Internal Medicine, Bundang Jaseng Hospital of Korean Medicine, Gyeonggi-do, Republic of Korea; d Department of Rehabilitation Medicine, Bundang Jaseng Hospital of Korean Medicine, Gyeonggi-do, Republic of Korea.

**Keywords:** integrative Korean medicine, older population, retrospective study, traffic accident

## Abstract

With the rapid aging of its population, Korea is becoming a super-aged society, and the proportion of older individuals among traffic accident patients continues to rise. Older adults often exhibit delayed recovery after trauma, multimorbidity, and polypharmacy, necessitating a tailored treatment approach. Integrative Korean medicine (IKM) treatment may serve as a suitable alternative, and its effectiveness and safety in older adults involved in traffic accidents warrant further evaluation. This retrospective chart review assessed the therapeutic efficacy and safety of IKM in hospitalized traffic accident patients aged 65 years or older, using electronic medical records from 4 branches of Korean medicine hospitals between 2021 and 2023. A total of 1788 patients were included in the analysis. Descriptive analyses were performed to summarize demographic and clinical characteristics, and within-group comparisons between admission and discharge were conducted for primary outcomes, including pain, quality of life, functional disability, and range of motion. Safety was assessed based on adverse events (AEs). A total of 1788 older inpatients with traffic-related injuries showed significant improvements (*P* < .001) in pain numeric rating scale (NRS), quality of life (European quality of life – 5 dimensions), functional disability indicators (neck disability index, Oswestry disability index, shoulder pain and disability index, Western Ontario and McMaster Universities osteoarthritis index), and range of motion after receiving IKM treatment. Specifically, the mean neck NRS score showed a reduction from 5.17 ± 0.93 at admission to 3.49 ± 1.24 at discharge, and the mean lower back NRS score improved from 5.19 ± 0.91 to 3.55 ± 1.21. Most AEs were mild, and there were no reports of serious AEs. IKM may be a viable and safe therapeutic approach to improving pain, function, and quality of life in older patients hospitalized after traffic accidents.

## 1. Introduction

According to Article 54, Paragraph 1 of the ‘Korean Road Traffic Act,’ “traffic accident” refers to “an incident in which a person is injured or killed, or property is damaged, due to traffic-related activities such as vehicle operation.” The term “vehicle” encompasses “automobiles, motorcycles, and bicycles” as defined in Article 2 of the same Act.^[1]^ Based on data from the Korea Road Traffic Authority’s Traffic Accident Analysis System, 196,349 motor vehicle accidents occurred in Korea in 2024, equating to a daily average of approximately 536 incidents. The number of nonfatal injuries resulting from these accidents was 278,482, of which 44,564 (approximately 16%) involved individuals aged 65 years or older. This trend highlights the growing medical demand related to traffic accidents among the older population, and this proportion is expected to increase further with continued population aging.^[2]^

According to the “2024 Statistics on the Older adults,” published by Statistics Korea on September 26, 2024, the population aged 65 years and over has reached 9.938 million, accounting for 19.2% of the total population, and this figure is expected to rise, surpassing 40% by 2050.^[3]^ Meanwhile, the United Nations’ “Asia-Pacific Report on Population Ageing 2022” classifies societies based on the proportion of people aged 65 years or older: as aging society (7–14%), aged society (15–20%), and super-aged society (>21%).^[4]^ Korea, currently categorized as an aged society owing to declining birth rates and increasing life expectancy, is forecasted to advance to a super-aged society by 2025. This demographic shift is likely to affect not only the incidence of traffic accidents but also the demand for post-accident medical care and treatment strategies. In this context, the importance of individualized treatment approaches tailored to the physiological characteristics of older patients is becoming increasingly evident.

According to nationally certified statistics from the Healthcare Big Data Open System as of July 2024, 2249,789 patients received medical treatment at various healthcare institutions (including tertiary hospitals, general, hospitals, long-term care hospitals, psychiatric hospitals, clinics, Korean medicine hospitals, and Korean medicine clinics) following traffic accidents in 2023.^[5]^ The total medical expenditure reached approximately KRW 2.56 trillion, with an average cost per case of KRW 128,556 and a per capita cost of KRW 1138,566. Among these patients, 1628,927 (72.4%) received treatment at Korean medicine hospitals or clinics, accounting for more than half of the total medical expenditure (KRW 1.32 trillion, 51.67%). This indicates a substantial reliance on Korean medical institutions to manage post-accident symptoms. Age-specific data revealed that 3285,554 treatment claims were filed by individuals aged 60 to 69 years and 1,611,231 claims by those aged 70 years or older: together representing 24.6% of all claims in 2023. These outcomes demonstrate that Korean medicine plays a significant role in the treatment of older patients following traffic accidents, reflecting its high utilization rate among older adults.

Despite this trend, only a few studies have specifically evaluated the effectiveness and safety profiles of Korean medicine treatments in the older population. While certain studies have reported the efficacy and patient satisfaction with Korean medicine for traffic accident injuries,^[6–8]^ only a few have addressed age-specific injury patterns and differential treatment responses. Kong et al^[9]^ analyzed the Korea in-depth traffic accident study database and reported that the rate of serious injuries among older patients involved in non-catastrophic traffic accidents was 1.6-fold that of younger patients. They also noted age-related differences in the distribution of injury sites; head, neck, and abdominal injuries were more common in older adults, whereas chest, head, and neck injuries were predominant in younger individuals. Given that the severity and patterns of injury in older individuals involved in traffic accident differ from those in younger individuals, these findings underscore the need for more cautious and specialized treatment strategies. Within real-world medical practice, mild-to-moderate injuries that do not require surgery are more frequently encountered, often resulting in persistent pain and functional impairments. Considering this clinical reality, there is a growing need to develop conservative treatment strategies suitable for older patients and rigorously evaluate their safety and efficacy.

Owing to age-related conditions such as decreased muscle strength and osteoporosis, the older adult population is more susceptible to fractures, falls, and long-term complications following traffic accidents.^[10,11]^ Additionally, the presence of multiple chronic comorbidities increases the risk of complications if injuries are not properly managed.^[12]^ Polypharmacy and reduced liver and kidney functions can further limit the use of conventional medications, including analgesics, necessitating alternative therapeutic approaches that differ from those used for non-older patients.^[13]^ Moreover, older patients often experience prolonged symptoms and face physical and social barriers hindering active treatment. Given these characteristics, a comprehensive, individualized treatment strategy is essential. Integrative Korean medicine (IKM) (which addresses not only pain reduction but also functional recovery and overall systemic health) may offer a suitable alternative for older patients involved in traffic accidents. Thus, this study aimed to conduct a retrospective chart review that reflected real-world clinical settings to generate clinically meaningful, evidence-based data on the efficacy and safety of Korean medicine treatments for older patients, ultimately enhancing their clinical applicability.

Several clinical studies have reported favorable outcomes and safety of IKM. Park et al^[14]^ conducted a retrospective chart review of 50 patients with acute low back pain following traffic accidents. The study found significant reductions in lumbar pain intensity and improvements in functional disability, with no serious adverse events (AEs) reported. Considering that commonly used analgesics and muscle relaxants for post-accident low back pain can cause side effects such as drowsiness, dizziness, and diarrhea, IKM may represent a safer 1st-line treatment option. In addition, Son et al^[15]^ performed a randomized controlled pilot study involving 50 patients to evaluate the effects of pharmacopuncture administered alongside IKM treatment on psychological stress after traffic accidents, and significant improvements were observed in anxiety and depression scores. Collectively, comprehensive IKM interventions (including acupuncture, pharmacopuncture, cupping, moxibustion, and Chuna manual therapy) may alleviate both musculoskeletal and psychological symptoms, thereby supporting recovery from the diverse consequences of traffic accidents.

Accordingly, this research sought to evaluate the clinical outcomes and safety profile of IKM in traffic accident patients aged 65 years and older, based on electronic medical records (EMRs).

## 2. Materials and methods

### 2.1. Study design and procedures

This retrospective chart review was carried out on patients hospitalized for treatment at 4 branches of the Jaseng Hospital of Korean Medicine (Gangnam, Bucheon, Daejeon, and Busan in South Korea) following traffic accident-related injuries between January 1, 2021, and December 31, 2023. This study was approved by the Institutional Review Board of the Jaseng Hospital of Korean Medicine (Institutional Review Board approval number: JASENG 2024-07-019) and was conducted in accordance with the relevant ethical guidelines and regulations. The study was registered at ClinicalTrials.gov (registration number: NCT07007299) on June 11, 2025 (Detailed registration information is publicly accessible at https://register.clinicaltrials.gov/prs/beta/studies/S000FR0Q00000047/recordSummary). This retrospective chart review followed the Strengthening the Reporting of Observational Studies in Epidemiology checklist for observational studies to ensure transparent reporting of study design, patient selection, and outcome assessment.^[16]^ As this was a retrospective analysis of previously administered treatments, no additional interventions were performed, and all patient data were obtained through a medical record review. Personal identification information was protected in accordance with privacy regulations. No identifiable information was collected or recorded; instead, the data were anonymized by assigning unique serial numbers and analyzed in a de-identified format. This retrospective chart review included an analysis of clinical and demographic characteristics, including chief complaints and diagnoses related to traffic accidents; duration of hospitalization; past medical and social history; AEs during hospitalization; quality of life measured using the European quality of life: 5 dimensions (EQ-5D); functional status assessed using the Oswestry disability index (ODI), neck disability index (NDI), shoulder pain and disability index (SPADI), and Western Ontario and McMaster Universities osteoarthritis index (WOMAC); and range of motion (ROM) evaluated at both admission and discharge.

### 2.2. Participants

#### 2.2.1. Inclusion criteria

Complete enumeration was conducted across the 4 branches, and study participants were selected in accordance with the following inclusion criteria:

(i) age 65 years or older at the time of initial visit;
(ii) hospitalization at the study site for symptoms resulting from a traffic accident, during which the patient received at least 1 session of IKM treatment.

#### 2.2.2. Exclusion criteria

Exclusion criteria comprised the following:

(i) patients who received only consultation services or conventional (Western) medical treatment at the study site;
(ii) patients who received only outpatient care without hospitalization;
(iii) patients deemed unsuitable for study participation at the discretion of the investigators.

### 2.3. Interventions

This study focused on patients who received IKM treatment, including acupuncture, cupping, pharmacopuncture, herbal medicine, Chuna manual therapy, moxibustion, and physical therapy (interferential current therapy). Specific treatment modalities were chosen by clinicians depending on individual patient characteristics during routine clinical practice. Data were obtained based on the treatment records documented in the EMRs, including the types of treatments administered, number of treatment sessions, and detailed treatment components.

At the Jaseng Hospital of Korean Medicine, a standardized treatment protocol is followed for patients suffering from traffic-accident-related injuries; however, additional or alternative interventions may be administered according to the patient’s condition and the attending physician’s clinical judgment.

#### 2.3.1. Acupuncture treatment

Sterile disposable stainless-steel needles (Dongbang Acupuncture Inc., Korea; 0.25 × 30 mm, 0.30 × 40 mm, 0.25 × 40 mm, and 0.20 × 30 mm) were used for acupuncture. Treatment was administered to the patient in the prone position, and needles were typically retained for approximately 10 minutes per session, with adjustments made according to individual patient needs. The commonly used acupuncture points for neck pain include GB20, GB21, LI4, and BL10.^[17]^ For lower back pain, the frequently used points include SI3, BL62, BL60, BL40, CV4, BL23, and BL52.^[18]^

#### 2.3.2. Pharmacopuncture treatment

Pharmacopuncture was administered using a disposable syringe (Seongshim Medical, 1 mL, 29G × 1/2 syringe), with a standard injection volume of 1.0 cc, although the actual volume varied depending on the patient. The primary pharmacopuncture solution used for musculoskeletal disorders at the study site was Shinbaro 2, which contains a combination of herbal ingredients such as Eucommiae Cortex, Acanthopanacis Cortex, Angelicae Pubescentis Radix, Saposhnikoviae Radix, Achyranthis Radix, Cervi Cornu Parvum, Paeoniae Radix, and others. This formulation is known for its anti-inflammatory properties, pain-relieving effects, and its potential to promote the regeneration of damaged bone and nerve tissues.^[19]^

#### 2.3.3. Herbal medicine treatment

Herbal medicines were prescribed based on the individual condition of each patient, using formulations prepared by the external herbal dispensary of the Jaseng Hospital of Korean Medicine. Anshinjitong-tang, an herbal formula designed to alleviate post-accident sequelae, is commonly prescribed for traffic accident patients. It contains ingredients such as Persicae Semen and Carthami Flos, which are believed to aid recovery from contusions and relieve pain, as well as Zizyphi Semen, Polygalae Radix, Ginseng Radix, and Astragali Radix, which are thought to promote mental and physical relaxation.^[20]^

#### 2.3.4. Chuna manual therapy

Chuna manual therapy is a treatment method that involves manipulating specific areas of the body, such as tender points on the superficial fascia and joints throughout the body, using manual techniques to regulate physiological and pathological conditions within the body. Chuna therapy is clinically used for peripheral nervous system disorders and pain-related conditions of the spinal and joint regions.^[21]^

#### 2.3.5. Physical therapy

Cupping therapy was applied around the areas of pain. Interferential current therapy, a treatment method that utilizes interferential currents generated by the crossing of 2 medium-frequency currents to stimulate deep muscles, was administered. Additionally, electromoxibustion therapy was used to provide a warming effect on painful areas, promoting blood circulation and relaxing local muscles.^[22]^

### 2.4. Outcomes

To ensure standardization and consistency of the measurements, all assessments and surveys were conducted by experienced Korean medicine doctors.

#### 2.4.1. Primary outcome

Patient’s subjective pain levels were measured using a numeric rating scale (NRS). The NRS is a widely used tool for assessing pain, in which patients select a number between 0 and 10 to represent the intensity of their pain. The NRS is simple to administer, applicable regardless of the patient’s age, and functions as an interval scale with equal intervals between numbers, allowing for parametric statistical analyses. Additionally, the National Institutes of Health Pain Consortium recommends it as a reliable measure in both clinical and research settings.^[23]^ The NRS scores were recorded at the points of admission and discharge at the anatomical sites corresponding to the patient’s chief complaints.

#### 2.4.2. Secondary outcomes

The overall quality of life of the patients was measured using the EQ-5D instrument. The EQ-5D assesses the health-related quality of life across 5 dimensions: mobility, self-care, usual activities, pain/discomfort, and anxiety/depression. Each dimension was rated on a 5-point scale: no problem, slight, moderate, severe, and extreme problems.^[24]^ There is an inverse relationship between the score and the patient’s quality of life; lower scores signify poorer quality. The scores were recorded at admission and discharge. Respondents provided a 5-level response for each dimension, and this combination describes the patient’s health status, referred to as the descriptive profile. The descriptive profile was transformed into a single summary index score using the value sets developed for each country. According to the Korean EQ-5D-5L Value Set, full health was assigned a value of 1 with a deduction of 0.096 for any deviation from full health. Roudijk et al^[25]^ described varying deductions based on scores in each dimension. Index scores in this study were calculated using this method.

NDI was used to assess the cervical functional disability. The NDI comprises 10 items, each scored on a 6-point scale ranging 0 to 5.^[7]^ In this study, the official Korean version of the NDI questionnaire was used. Higher scores indicate greater disability. The scores were obtained at admission and discharge.

The ODI was used to evaluate the lower back pain symptoms. It comprises 10 items related to activities of daily living (pain intensity, sleeping, self-care, walking, sitting, standing, lifting, sexual activity, social life, and traveling), with patients rating each item on a scale 0 to 5.^[26]^ Higher scores indicate more severe functional impairment. Measurements were performed at admission and discharge.

Additional questionnaires were administered to the patients who reported shoulder or knee symptoms. Shoulder function was evaluated using the SPADI, which assesses 2 subdomains: pain and disability. The patients rated each item using an integer score ranging from 0 (no pain) to 10 (worst pain).^[27]^ Knee function was assessed using the WOMAC index, which includes 3 subdomains: pain, stiffness, and physical function, with responses scored from 1 to 5.^[7]^ Both questionnaires interpret higher scores as worse joint function. Scores were recorded at admission and discharge.

ROM assessments were performed at admission and discharge. Normal values for cervical ROM were defined as flexion of 45°, extension of 45°, right (R)/left (L) lateral bending of 45°/45°, and R/L rotation of 90°/90°. Normal lumbar ROM values were flexion of 90°, extension of 20°, R/L lateral bending of 30°/30°, and R/L rotation of 45°/45°. Normal shoulder ROM values were R/L flexion of 160°/160°, R/L extension of 45°/45°, R/L abduction of 170°/170°, R/L adduction of 45°/45°, R/L internal rotation of 80°/80°, and R/L external rotation of 45°/45°. The normal knee ROM values were R/L flexion of 135°/135°, R/L extension of 0°/0°, R/L internal rotation of 5°/5°, and R/L external rotation of 5°/5°. If any ROM parameter measured at admission or discharge was below the normal range, that time point was classified as ‘abnormal.’ Patients with missing data for all ROM parameters were excluded. Patients with at least 1 abnormal ROM measurement was classified into the “abnormal ROM group” and the number of patients exhibiting limited ROM was recorded and analyzed.

#### 2.4.3. Safety assessment

AEs were comprehensively investigated for any symptoms of discomfort reported by patients at least once during hospitalization. The investigation included the type of AE, resolution status during hospitalization, severity of the AE, presence of serious AEs, interventions undertaken in response to the AE, causality assessment between the AE and IKM treatment, and outcomes following the AE. The classification of AE severity followed Spilker criteria as detailed below^[28]^: mild, no treatment required and minimal interference with the patient’s normal daily function; moderate, significant interference with normal function, possibly requiring treatment with recovery after intervention; severe, serious AEs requiring intensive treatment and potentially resulting in sequelae.

The causality between each AE and the IKM treatment was assessed using the World Health Organization – Uppsala Monitoring Centre system for standardized case causality assessment, which categorizes causality into 6 groups: certain; probable/likely; possible; unlikely; conditional/unclassified; and unassessable/unclassifiable.^[29]^ Two independent researchers conducted the causality assessments, and discrepancies were resolved through consensus with a 3rd reviewer.

### 2.5. Statistical analyses

This study used retrospective medical records as data source. Descriptive analyses were conducted to summarize the traffic accident characteristics and demographic information. Continuous variables are summarized using mean ± standard deviation (SD), and categorical variables are described by frequency and percentage. All efficacy evaluation variables (NRS, NDI, ODI, SPADI, WOMAC, and EQ-5D) were assessed based on the differences in scores between admission and discharge. The normality of the change scores was evaluated using the Shapiro–Wilk test. Considering that this test can be influenced by sample size, skewness, and kurtosis values were used as supplementary indicators along with the *P*-values.^[30]^ Variables that satisfied the normality assumption were analyzed using a paired *t* test, whereas variables that did not satisfy normality were analyzed using the Wilcoxon signed-rank test. Statistical analyses were performed with R software (version 4.1.3; R Foundation for Statistical Computing, Vienna, Austria).

## 3. Results

### 3.1. Participants

A retrospective chart review was conducted for patients who received inpatient treatment at 4 branches of Jaseng Hospital of Korean Medicine. Based on the inclusion and exclusion criteria, a total of 1788 patients were enrolled. Patient baseline characteristics are detailed in Table 1. Patients’ demographics were characterized by: mean age was 72.96 ± 4.77 years, and the sample included 941 males and 847 females. Among 1134 patients whose height was measured, the average height was 161.30 ± 8.08 cm, and for the same number of patients whose weight was recorded, the mean weight was 62.38 ± 9.83 kg. The average length of hospital stay was 9.94 ± 6.06 days. Regarding prior interventions before hospitalization, 41 patients (2.29%) had received only Korean medicine treatments, and only 4 patients (0.22%) had received both Korean and conventional medical treatments. Given the traits of the older population, the majority of patients (65.25%) were unemployed, followed by those engaged in service and sales occupations (12.05%), and clerical workers (4.65%).

**Table 1:** Baseline characteristics.

Variable	Total (n = 1788) (mean ± SD or number(%))	
Age	72.96 ± 4.77	
Sex	Male	941 (52.63%)
	Female	847 (47.37%)
Height (n = 1134)	161.30 ± 8.08	
Weight (n = 1134)	62.38 ± 9.83	
Length of stay	9.94 ± 6.06	
Pre-admission intervention	No intervention	1220 (68.23%)
	Korean medicine (KM) – only intervention	41 (2.29%)
	Conventional medicine (CM) – only intervention	523 (29.25%)
	Combined Korean and conventional medicine (KM + CM) intervention	4 (0.22%)
Smoking (n = 1774)	Former smoker	59 (3.33%)
	Current smoker	122 (6.88%)
	Never smoker	1593 (89.80%)
Drinking	Yes	265 (14.82%)
	No	1523 (85.18%)
Job (n = 1784)	Unemployed (including homemakers and students)	1164 (65.25%)
	Managerial position	55 (3.08%)
	Professionals and related workers	79 (4.43%)
	Clerical workers	83 (4.65%)
	Service and sales workers	215 (12.05%)
	Skilled agricultural, forestry, and fishery workers	34 (1.91%)
	Craft and related trades workers	30 (1.68%)
	Plant, machine operators, and assemblers	13 (0.73%)
	Elementary occupations	110 (6.17%)
	Armed forces personnel	1 (0.06%)

Data are represented as either mean ± standard deviation (SD) or number (%).

**Figure 1.**
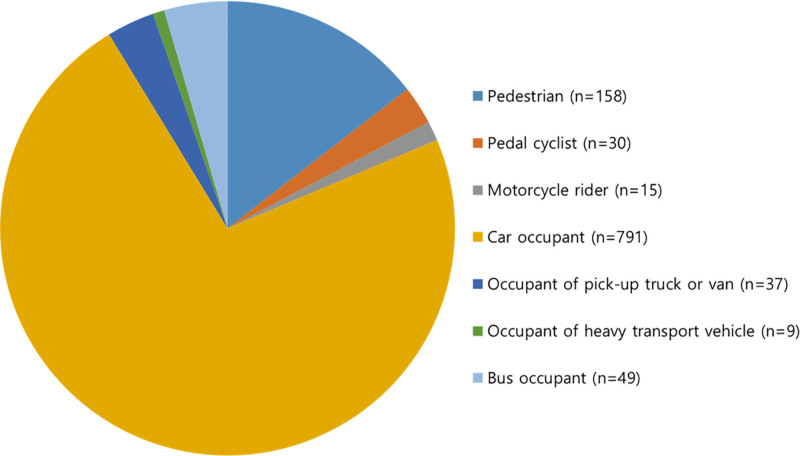
Types of transport accident participants (n = 1089).

The most frequent primary diagnoses recorded in the medical charts were analyzed and are presented in Table 2. Based on the Korean Standard Classification of Diseases and Causes of Death, the most common diagnoses were S3350 – sprain and strain of lumbar spine (1238 cases, 69.24%), S134 – sprain and strain of cervical spine (807 cases, 45.13%), and M518 – other specified intervertebral disc disorders (117 cases, 9.9%). Further, to understand the nature of traffic accidents, a detailed analysis was performed on the V-codes, which indicated transport accident-related external causes of injury. Among the 1089 V-code entries, the most frequent diagnoses were V435 and V436 – car driver and passenger, respectively, injured in collision with car, pick-up truck or van in traffic accident (305 cases, 28.01%) (see Table S1, Supplemental Digital Content, https://links.lww.com/MD/Q744, which illustrates the type of accidents and V-codes). When categorized by the type of transport accident, the most common was car occupants (791 cases), and the least common was heavy truck occupants (9 cases) (Fig. 1).

**Table 2:** Top 10 most frequent principal diagnosis.

Principal diagnosis	Frequency	Percentage of cases (1788)
S3350	1238	69.24
S134	807	45.13
M518	177	9.9
S434	173	9.68
M508	155	8.67
S836	80	4.47
S235	50	2.8
S7319	42	2.35
S6359	39	2.18
S0600	35	1.96

S3350 = sprain and strain of lumbar spine; S134 = sprain and strain of cervical spine; M518 = other specified intervertebral disc disorders; S434 = sprain and strain of shoulder joint; M508 = other cervical disc disorders; S836 = sprain and strain of other and unspecified parts of knee; S235 = sprain and strain of other and unspecified parts of thorax; S7319 = sprain and strain of unspecified site of hip; S6359 = sprain and strain of wrist, part unspecified; S0600 = concussion, without open intracranial wound.

The most common underlying diseases were hypertension (859 patients, 48.04%), followed by cardiovascular diseases, and diabetes. A total of 773 patients (43.23%) had a history of surgery and 178 (9.96%) had a history of fractures (Table 3).

**Table 3:** Comorbidity of patients.

Comorbidity	Total (n = 1788)	
Hypertension	Yes	859 (48.04%)
	No	929 (51.96%)
Diabetes mellitus	Yes	450 (25.17%)
	No	1338 (74.83%)
Respiratory	Yes	125 (6.99%)
	No	1663 (93.01%)
Cardiovascular	Yes	737 (41.22%)
	No	1051 (58.78%)
Brain-nervous	Yes	85 (4.75%)
	No	1703 (95.25%)
Gastrointestinal	Yes	261 (14.60%)
	No	1527 (85.40%)
Surgery	Yes	773 (43.23%)
	No	1015 (56.77%)
Past fracture	Yes	178 (9.96%)
	No	1610 (90.04%)

### 3.2. Treatment

An analysis of the specific treatment components and number of treatment sessions per patient revealed that 1787 patients (99.94%) received acupuncture during hospitalization, typically twice a day. Pharmacopuncture was administered to 1781 patients (99.61%), with an average of 6.45 ± 2.82 sessions during their hospitalization. Herbal medicine was administered to 1719 patients (96.14%), with a mean number of 43.68 ± 20.01 doses. Additionally, 1594 patients (89.15%) received Chuna manual therapy, with an average of 7.73 ± 4.22 sessions. Among Korean medicine physiotherapies, 1786 patients (99.89%) received cupping therapy, and 1684 patients (94.18%) received moxibustion therapy. These results indicate that most patients received IKM treatments using various modalities (Table 4).

**Table 4:** Types of treatment.

Types of treatment	Total (n = 1788)	
Acupuncture	Yes	1787 (99.94%)
	No	1 (0.06%)
Pharmacopuncture	Yes	1781 (99.61%)
	No	7 (0.39%)
Herbal medicine	Yes	1719 (96.14%)
	No	69 (3.86%)
Chuna therapy	Yes	1594 (89.15%)
	No	194 (10.85%)
Cupping	Yes	1786 (99.89%)
	No	2 (0.11%)
Interference current therapy	Yes	1200 (67.11%)
	No	588 (32.89%)
Moxibustion	Yes	1684 (94.18%)
	No	104 (5.82%)

Among the 1781 patients who received pharmacopuncture, 1986 prescriptions were recorded. The most frequently prescribed pharmacopuncture solution was Shinbaro 2, as specified in the treatment protocol, which accounted for 1066 cases (53.68%). Other commonly used solutions included Hwangryunhaedok pharmacopuncture (532 cases, 26.79%), neutral-stasis pharmacopuncture (224 cases, 11.28%), and Shinbaro 1 pharmacopuncture (124 cases, 6.24%).

Among the 1719 patients who received herbal medicine, 2177 prescriptions were documented. The most frequently prescribed herbal formula was Anshinjitong-tang, as stated in the protocol, in 1229 cases (56.45%). This was followed by Hwalhyeoljitong-tang (395 cases, 18.14%) and a modified version of Gyulchulsamul-tang (199 cases, 9.14%) (see Table S2, Supplemental Digital Content, https://links.lww.com/MD/Q744, which presents the types of pharmacopuncture and herbal medicines).

### 3.3. Outcomes

**Figure 2.**
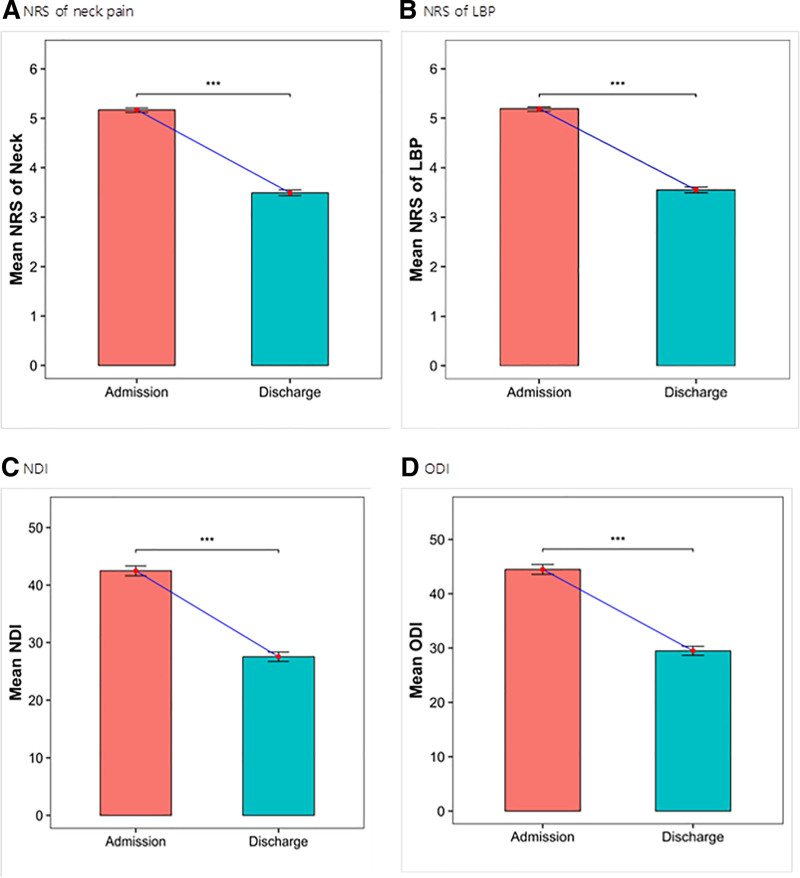
Changes in NRS scores and questionnaire outcomes for neck and lower back regions. NRS of neck pain (B) NRS of LBP (C) NDI (D) ODI. LBP = low back pain, NDI = neck disability index, NRS = numeric rating scale, ODI = the Oswestry disability index. *P*-values: ^***^P < .001.

**Figure 3.**
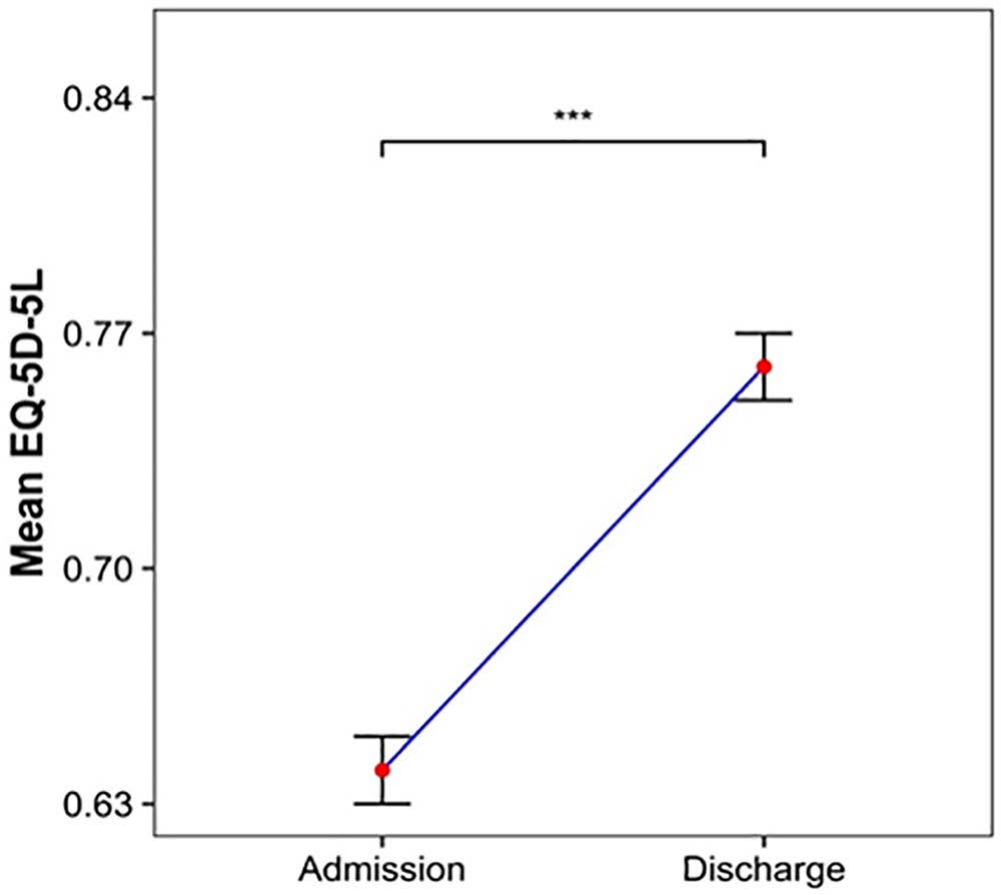
Changes in EuroQol 5 Dimensions – 5 Level. *P*-values: ^***^P < .001.

Primary outcome, the NRS, was measured based on the patients’ reported sites of pain, and only patients with both NRS scores recorded upon admission and at the time of discharge were included in the analysis. For shoulder and knee pain, values were recorded separately for the left and right sides, as some patients reported pain only on 1 side. For neck pain, NRS scores were available for 1615 patients, showing an improvement from a mean of 5.17 ± 0.93 at admission to 3.49 ± 1.24 at discharge. (*P* < .001, Fig. 2A). For lower back pain, NRS scores were recorded for 1628 patients and improved from a mean of 5.19 ± 0.91 at admission to 3.55 ± 1.21 at discharge. (*P* < .001, Fig. 2B). Additionally, both shoulder and knee pain showed statistically significant reductions from admission to discharge (*P* < .001), with detailed values presented in Table 5. The EQ-5D was administered to 1337 inpatients. The mean score improved from 0.64 ± 0.15 at admission to 0.76 ± 0.12 at discharge, indicating an enhancement in quality of life (*P* < .001, Fig. 3). The NDI was measured for 1206 patients at both admission and discharge, showing improvement from 42.48 ± 15.33 to 27.54 ± 14.50, respectively (*P* < .001). The ODI was measured for 1213 patients and improved from 44.49 ± 16.24 at admission to 29.48 ± 14.78 at discharge (*P* < .001). In addition, other joint function-related assessments such as the SPADI and WOMAC for knees also demonstrated statistically significant improvement between admission and discharge (*P* < .001; Table 5).

**Table 5:** Numeric rating scale and questionnaire outcomes.

		n	Mean	SD	*P*	Mean of the difference* (95% CI)
NRS of neck pain	Admission	1615	5.17	0.93	<.001	−1.68 (−1.74 to −1.62)
	Discharge	1615	3.49	1.24		
NRS of LBP	Admission	1628	5.19	0.91	<.001	−1.64 (−1.69 to −1.58)
	Discharge	1628	3.55	1.21		
NRS of shoulder pain (right)	Admission	284	4.51	1.29	<.001	−1.76 (−1.92 to −1.61)
	Discharge	284	2.75	1.60		
NRS of shoulder pain (left)	Admission	268	4.55	1.32	<.001	−1.75 (−1.91 to −1.60)
	Discharge	268	2.79	1.63		
NRS of knee pain (right)	Admission	225	4.95	1.16	<.001	−1.75 (−1.93 to −1.57)
	Discharge	225	3.20	1.46		
NRS of knee pain (left)	Admission	217	4.97	1.14	<.001	−1.71 (−1.90 to −1.53)
	Discharge	217	3.26	1.50		
EQ-5D	Admission	1337	0.64	0.15	<.001	0.12 (0.11 to 0.13)
	Discharge	1337	0.76	0.12		
NDI	Admission	1206	42.48	15.33	<.001	−14.94 (−15.79 to −14.08)
	Discharge	1206	27.54	14.50		
ODI	Admission	1213	44.49	16.24	<.001	−15.01 (−15.86 to −14.15)
	Discharge	1213	29.48	14.78		
SPADI	Admission	162	41.34	20.38	<.001	−11.58 (−14.60 to −8.56)
	Discharge	162	29.76	18.98		
WOMAC	Admission	141	67.37	16.70	<.001	−15.00 (−17.65 to −12.35)
	Discharge	141	52.37	18.36		

EQ-5D = European Quality of Life – 5 Dimension, LBP = low back pain, NDI = neck disability index, NRS = numeric rating scale, ODI = the Oswestry disability index, SPADI = shoulder pain and disability index, WOMAC = Western Ontario McMaster.

* Discharge–Admission.

The number of patients presenting with abnormal ROM also decreased significantly across all joints at discharge, suggesting functional improvement. However, it should be noted that pain does not necessarily result in passive ROM limitations, and these figures may not fully reflect the total number of assessed patients assessed (see Table S3, Supplemental Digital Content, https://links.lww.com/MD/Q744, which presents the changes in patients with limited ROM).

### 3.4. Safety

During hospitalization, any discomfort reported by patients outside their chief complaints was recorded as an AE by the ward nurses and Korean medicine doctors. These records were extracted from EMR and analyzed, as summarized in Table 6. Among the 1788 patients, 326 (18.23%) reported 537 AEs.

**Table 6:** Types of adverse events.

Types of adverse events	Case
	n (% of total cases of adverse events)
Dyspepsia	117 (21.79)
Upper respiratory symptoms	98 (18.25)
Insomnia	82 (15.27)
Headache	77 (14.34)
Constipation	60 (11.17)
Diarrhea	26 (4.84)
Anxiety	20 (3.72)
Fatigue	17 (3.17)
Body ache	17 (3.17)
Sweating	4 (0.74)
Dry mouth	4 (0.74)
Muscle cramps	3 (0.56)
Fever	3 (0.56)
Urinary symptoms	3 (0.56)
Pruritus	2 (0.37)
Stomatitis	1 (0.19)
Rhinitis	1 (0.19)
Anorexia	1 (0.19)
Tinnitus	1 (0.19)
Total	537 (100.00)

Among these, dyspepsia was the most frequently reported AE, accounting for 117 cases (21.79%), followed by upper respiratory symptoms, insomnia, and headache. Most AEs were mild. In cases where AEs occurred, most patients were managed with appropriate medication (492 cases, 91.62%). No specific intervention was required in 32 (5.96%) patients. Hospitalization or extension of hospital stay was needed in 4 cases (0.74%), and therapeutic or diagnostic procedures were performed in 8 cases (1.49%). One case (0.19%) was categorized as other. Consequently, 415 patients (77.28%) recovered during hospitalization with observation and medication, whereas the remaining 122 patients (22.72%) were still recovering at the time of discharge.

Evaluation of the causal relationship between these AEs and the IKM treatment revealed that none of the cases were classified as having a ‘certain’ causal relationship. Only 4 cases (0.74%) were assessed as “probable,” and 12 cases (2.23%) as ‘possible.’ The remaining 521 cases (97.02%) were considered unlikely to be related to or have an undetermined association with IKM treatment. However, some cases of diarrhea or digestive discomfort are presumed to be associated with herbal medicine intake. Importantly, there were no reports of serious AEs such as death, life-threatening conditions, permanent or significant physical disabilities or impairments.

## 4. Discussion

This study examined the clinical efficacy and safety profile of IKM treatment through a retrospective analysis using real-world data of 1788 older patients aged ≥ 65 years who were hospitalized due to traffic accidents at our institution. According to the Health Insurance Review & Assessment Service’s automobile insurance medical expense statistics, from 2019 to 2024, musculoskeletal disorders accounted for the highest frequency of diagnoses among patients visiting Korean medicine institutions following automobile accidents, both for inpatient and outpatient care. Specifically, the most frequent diagnoses were: S13, S33, and S43, relating to dislocation, sprain, and strain of the joints and ligaments in the neck region, lumbar spine and pelvis, and shoulder girdle, respectively. Most top-ranking conditions were musculoskeletal disorders, highlighting that many patients visit Korean medicine institutions for various musculoskeletal injuries resulting from automobile accidents. This indicates that Korean medicine institutions are instrumental in the treatment of musculoskeletal injuries in traffic accident patients.^[2]^

Meanwhile, the age of patients involved in traffic accidents has been increasing. Lee et al^[31]^ conducted the cognitive perceptual assessment for driving test (CPAD) on 168 older drivers aged ≥ 65 years to examine the association between cognitive and perceptual functions and traffic accident occurrence. The group with a history of accidents scored lower on the CPAD than did the group without such a history, but the difference did not reach statistical significance. In contrast, the group aged 70 years and older showed a higher rate of traffic accidents and significantly lower CPAD scores than the 65 to 69 years age group. This suggests a general decline in perceptual ability, sustained attention, cognitive function, and physical capability in older drivers. Additionally, Kim et al^[32]^ conducted an analysis of 1172 male drivers aged 60 years or older using data from the Korean longitudinal study on cognitive aging and dementia, and found that driving-related AEs increased notably in those in their late 80s, with higher memory test scores significantly associated with a lower risk of dangerous driving (*P* = .039). Similarly, Lee et al^[33]^ analyzed various factors affecting traumatic brain injury (such as vehicle type, seating position, and seatbelt use) in passengers aged over 55 years. These issues regarding traffic accident in older patients are expected to persist as the society ages. Moreover, older patients tend to experience physiological changes owing to aging, coexistence of chronic diseases, polypharmacy, and reduced drug metabolism, which often impose limitations on conventional drug treatments and active therapeutic approaches. Additionally, although immediate surgery is often not required for older patients with traffic accidents, injuries affecting daily function are frequently observed, and symptoms tend to become chronic owing to reduced recovery capacity. Considering the treatment limitations and physiological characteristics of older patients, Korean medicine (which is noninvasive and low-risk) offers a suitable, integrated approach that supports pain relief, functional recovery, and safer medication management. However, research evaluating the efficacy and safety of traditional Korean medicine treatments, specifically for older patients involved in traffic accidents, remains insufficient. In a previous study, Lee et al^[34]^ retrospectively analyzed EMRs from 3 Korean medicine hospitals to understand the characteristics of Korean medicine use in traffic accident patients. The study included 384 patients who continued treatment for more than 3 months after the accident, with an average age of 50 years, and presented data on accident types and treatment modalities. However, as this study included patients across all age groups, it was difficult to analyze the characteristics and treatment effects specific to older patients. Additionally, the relatively small sample size and limited data on the sole efficacy measure, the NRS, limited the representativeness of the findings.

The study population of older adults had an average age of about 73 years, with sex distribution approaching equilibrium. Despite their advanced age, the patients were within a relatively standard body size range, with an average height and weight. The average duration of hospitalization was 9.94 ± 6.06 days, approximately 10 days. These findings should be considered in light of potential limitations on hospitalization duration under automobile insurance, and the possibility that patients may continue treatment through outpatient care if their pain is not fully resolved at discharge. Regarding prior interventions before treatment, most patients visited directly without any prior intervention, while a larger proportion received conventional medical care alone compared with Korean medicine alone. It is presumed that many patients visited conventional medical institutions immediately after the accident for examination, and subsequently sought Korean medical care for further treatment.

Analysis of the patients’ primary diagnoses and clinical characteristics revealed that cervical and lumbar sprains or strains were the most common conditions, reflecting the prevalence of musculoskeletal injuries typically observed following traffic accidents.^[35]^ Regarding comorbidities and past medical history, hypertension and cardiovascular disease were highly prevalent, and a considerable number of patients had diabetes. In addition, a notable proportion of patients had a history of surgery (43.23%) or fractures (9.96%). These findings highlight the importance of comprehensive management of underlying chronic diseases, along with musculoskeletal injury treatment in clinical care and rehabilitation planning for older patients involved in traffic accidents.

Among patients with available NRS data, statistically significant reductions in pain intensity were observed across 6 body regions (neck, lower back, left shoulder, right shoulder, left knee, and right knee) following IKM treatment compared to those at admission (*P* < .001). These findings suggest that IKM may be effective for pain relief. Farrar et al^[36]^ reported a consistent correlation between changes in the NRS and the patient global impression of change across various disease types, age groups, sexes, study outcomes, and treatment arms. A reduction of approximately 2 points or 30% on the NRS is widely accepted as the minimum clinically important difference (MCID). In the present study, the changes in NRS scores met or exceeded this MCID threshold across all regions: the neck NRS decreased by 1.68 points (from 1.62–1.74; 32.49% reduction), lower back by 1.64 points (1.58–1.69; 31.60%), right shoulder by 1.76 points (1.61–1.92; 39.02%), left shoulder by 1.75 points (1.60–1.91; 38.46%), right knee by 1.75 points (1.57–1.93; 35.35%), and left knee by 1.71 points (1.53–1.90; 34.41%). The reductions in NRS indicate clinically meaningful improvements in pain levels, suggesting that the observed changes were not only statistically significant but also perceptible to the patients. These findings support the clinical utility of IKM for pain management.

Among patients for whom EQ-5D data were available, a statistically significant enhancement in health-related quality of life scores was observed at discharge compared with admission (*P* < .001), with an average increase of 0.12 points. This exceeds the MCID for the EQ-5D, which ranges from 0.040 to 0.082, indicating a clinically meaningful improvement in quality of life.^[37]^ The NDI showed a total change of 14.94 points (*P* < .001), surpassing the MCID range 7.5 to 10.5 points,^[38]^ indicating a significant improvement in cervical function. The ODI improved by 15.01 points (*P* < .001), which also exceeded the MCID threshold of approximately 10 points,^[39]^ indicating a clinically meaningful enhancement in lumbar function. The SPADI demonstrated a change of 11.58 points (*P* < .001), exceeding the MCID of 8 points,^[27]^ suggesting a clinically significant improvement in shoulder function. The WOMAC improved by 15.00 points (*P* < .001), exceeding the MCID threshold of 12 points,^[40]^ indicating a clinically meaningful improvement in lower extremity function.

In this study, a combination of various Korean medicine treatments: including acupuncture, pharmacopuncture, herbal medicine, and Chuna manual therapy, were administered. Wu et al reported that various forms of acupuncture demonstrated significant therapeutic effects in patients with acute lower back pain compared with placebo or conventional medication.^[41]^ Additionally, Jang et al suggested that acupuncture is not only effective in alleviating pain but also in improving emotional symptoms such as depression and anxiety, thereby offering multifaceted symptom relief for individuals suffering from chronic pain.^[42]^ In a randomized controlled trial by MacPherson et al, the therapeutic effects of acupuncture were sustained for up to 12 months, supporting its potential long-term therapeutic benefits.^[43]^ Kim et al described pharmacopuncture as a technique that enhances the effects of acupuncture by injecting refined herbal extracts into acupoints, with various outcomes depending on the components used. Pharmacopuncture is widely applied in multiple medical (fields, including gastrointestinal, circulatory, and respiratory disorders), and is particularly prominent in the field of rehabilitation medicine for musculoskeletal and neurological conditions.^[44]^ In particular, Shinbaro 2 pharmacopuncture, commonly used for spinal stenosis and herniated discs, has shown promising results in reducing neuroinflammation, improving motor function, and relieving pain.^[19,45]^ The herbal decoction, Anshinjitong-tang, included in the treatment protocol, is expected to alleviate symptoms such as headache, neck pain, and lower back pain by improving qi and blood circulation disrupted by traffic accidents.^[20]^ Furthermore, according to an umbrella review by Kim et al, Chuna manual therapy is considered as a well-tolerated and efficacious approach for managing diverse musculoskeletal conditions.^[46]^ Therefore, the IKM approach adopted in this study (based on the complementary effects of each modality) holds the potential to provide holistic therapeutic benefits for older patients involved in traffic accidents, addressing not only pain and functional impairments but also emotional well-being and overall health.

In this study, AEs were reported in 18.2% patients who received IKM treatment. However, most AEs were mild, and resolved during hospitalization. Among the reported AEs, 91.62% were appropriately managed with either Korean or conventional medical interventions, and cases requiring extended hospitalization or additional treatment were rare. No serious AEs were reported. Cases evaluated as having a “definite” or “probable” causal relationship with IKM accounted for only 0.74% of all AEs. Most of them were assessed as unlikely to be related to, or of uncertain causality. These findings suggest that IKM can be safely administered to older patients. Nonetheless, mild gastrointestinal symptoms, such as diarrhea or indigestion associated with herbal medicine intake, may occur depending on individual responses. Therefore, caution is advised during treatment, and future research should aim to establish a more systematic AE reporting framework and standardized criteria for causality assessment.

This study is subject to certain limitations inherent to its retrospective design. First, the analysis was based on information documented in the EMRs, which is inherent to retrospective data collection. Therefore, some clinical data may have been missing or inaccurately recorded, introducing potential information bias that could have affected the reliability of the study outcomes. Second, the interventions were implemented in real-world clinical settings, where treatment decisions were individualized based on the patient’s clinical status, preferences, and healthcare provider’s clinical discretion at the time of care. This lack of standardization in treatment protocols may have introduced heterogeneity, limiting the direct comparability and generalizability of treatment outcomes. Third, the study included patients with short-term hospitalization (≤3 days,) who may not have undergone a sufficient treatment course. Consequently, the effectiveness of IKM may have been underestimated, especially because short-term improvements may not reflect long-term clinical outcomes. Fourth, the study did not include a post-discharge follow-up, making it impossible to evaluate the lasting benefits of Korean medical intervention. Despite these limitations, this study offers significant clinical value as a real-world evidence investigation based on actual clinical data, focusing on patients aged 65 years or older: a population underrepresented in prior research. While previous studies have mostly focused on the demographic profiles and prognosis of traffic accidents in the older population, this study is academically valuable because it provides evidence on the therapeutic benefits and safety of IKM treatment in older patients who visited Korean medicine hospitals after traffic accidents. Additionally, the inclusion of a relatively large sample of 1788 patients enhances the reliability and objectivity of the findings. Further research is warranted to improve the standardization of interventions, ensure adequate pre- and posttreatment observation periods, and establish systematic AE reporting and causality assessment frameworks to generate more robust evidence on the safety and therapeutic value of IKM treatments.

## 5. Conclusion

This study offers an overview of older (≥65 years) patients involved in traffic accidents who underwent care at Korean medicine hospitals and demonstrate that IKM treatment may be an effective and safe therapeutic option. This study offers supporting evidence for considering such treatment as a potential future intervention. However, owing to limitations such as the lack of standardized interventions across patients and the inclusion of short-term inpatients, further research using prospective randomized controlled trials is warranted. Such studies would help standardize interventions and allow for a more precise evaluation of the therapeutic efficacy and safety of IKM treatments.

## Author contributions

**Conceptualization:** Ju-Yeun Shin, Su Won Lee.

**Data curation:** Ju-Yeun Shin, Seung-Hee Lee, Jeong-Hee Noh.

**Formal analysis:** Ye-Seul Lee.

**Investigation:** Ju-Yeun Shin.

**Supervision:** Yoon Jae Lee, In-Hyuk Ha, Ye-Seul Lee, Su Won Lee.

**Writing – original draft:** Ju-Yeun Shin.

**Writing – review & editing:** Ju-Yeun Shin, Su Won Lee.

## Supplementary Material


