# Supplementary material for: Real-world evidence on the safety and effectiveness of integrative Korean medicine for older patients post-traffic accident: A retrospective observational study
Source: Medicine (Baltimore). 2025 Nov 28;104(48):e46144. doi: 10.1097/MD.0000000000046144 (PMC12662537; doi:10.1097/MD.0000000000046144)
Supplement: Supplementary file 1 [file medi-104-e46144-s001.docx]

**Supplementary table 1.** Types of transport accident and V code (KCD)

| **Type of transport accident** | **type of V code** | **N (Total: 1089)** |
| --- | --- | --- |
| V02 Pedestrian injured in collision with two- or three-wheeled motor vehicle | V021 | 7 |
| V03 Pedestrian injured in collision with car, pick-up truck or van | V031 | 146 |
| V04 Pedestrian injured in collision with heavy transport vehicle or bus | V041 | 5 |
| V11 Pedal cyclist injured in collision with other pedal cycle | V117 | 1 |
| V12 Pedal cyclist injured in collision with two- or three-wheeled motor vehicle | V124 | 1 |
| V13 Pedal cyclist injured in collision with car, pick-up truck or van | V134 | 28 |
| V22 Motorcycle rider injured in collision with two- or three-wheeled motor vehicle | V224 | 2 |
| V23 Motorcycle rider injured in collision with car, pick-up truck or van | V234 | 13 |
| V42 Car occupant injured in collision with two- or three-wheeled motor vehicle | V425 | 5 |
|  | V426 | 1 |
| V43 Car occupant injured in collision with car, pick-up truck or van | V435 | 385 |
|  | V436 | 305 |
| V44 Car occupant injured in collision with heavy transport vehicle or bus | V445 | 46 |
|  | V446 | 33 |
| V47 Car occupant injured in collision with fixed or stationary object | V475 | 8 |
|  | V476 | 3 |
| V48 Car occupant injured in noncollision transport accident | V485 | 2 |
|  | V486 | 3 |
| V53 Occupant of pick-up truck or van injured in collision with car, pick-up truck or van | V535 | 24 |
|  | V536 | 10 |
| V54 Occupant of pick-up truck or van injured in collision with heavy transport vehicle or bus | V545 | 2 |
|  | V546 | 1 |
| V63 Occupant of heavy transport vehicle injured in collision with car, pick-up truck or van | V635 | 4 |
|  | V636 | 1 |
| V64 Occupant of heavy transport vehicle injured in collision with heavy transport vehicle or bus | V645 | 3 |
|  | V646 | 1 |
| V72 Bus occupant injured in collision with two- or three-wheeled motor vehicle | V726 | 1 |
| V73 Bus occupant injured in collision with car, pick-up truck or van | V736 | 15 |
| V74 Bus occupant injured in collision with heavy transport vehicle or bus | V746 | 5 |
| V78 Bus occupant injured in noncollision transport accident | V781 | 1 |
|  | V786 | 27 |

| **Supplementary table 2.** Types of pharmacopuncture and herbal medicine | | |
| --- | --- | --- |
| **(a)** Types of pharmacopuncture | | |
| **Types of pharmacopuncture** | **Frequency** | **Percentage of responses** |
| Shinbaro 2 | 1,066 | 53.68 |
| Hwangryunhaedok-tang pharmacopuncture | 532 | 26.79 |
| Jungsongouhyul phamacopuncture | 224 | 11.28 |
| Shinbaro 1 | 124 | 6.24 |
| Shinbaro 3 | 21 | 1.06 |
| Sweet bee venom | 9 | 0.45 |
| Jakyakgamcho Pharmacopuncture | 7 | 0.35 |
| Bee venom 5% | 3 | 0.15 |
| **Total** | **1,986** | **100.00** |
| **(b)** Types of herbal medicine |  |  |
| **Types of herbal medicine** | **Frequency** | **Percentage of responses** |
| Ansinjitong-tang | 1,229 | 56.45 |
| Hwalhyeoljitong-tang | 395 | 18.14 |
| Gyulchulsaengmul-tang Gagam | 199 | 9.14 |
| Others (16 types) | 354 | 16.26 |
| **Total** | **2,177** | **100.00** |

**Supplementary table 3.** Changes in patients with limited range of motion (ROM)

|  | Total number of patients  assessed for ROM | Number of patients with limited ROM^*^ | |
| --- | --- | --- | --- |
| Neck | 1,541 | Admission | 231 |
|  |  | Discharge | 67 |
| Lumbar | 1,529 | Admission | 221 |
|  |  | Discharge | 82 |
| Shoulder(R) | 320 | Admission | 45 |
|  |  | Discharge | 18 |
| Shoulder(L) | 318 | Admission | 44 |
|  |  | Discharge | 23 |
| Knee(R) | 262 | Admission | 13 |
|  |  | Discharge | 8 |
| Knee(L) | 261 | Admission | 13 |
|  |  | Discharge | 7 |

^*^Regarded as limited ROM if even one of the ROM assessment items for the corresponding joint fell outside the normal range
